# Phospholipid-mimicking block, graft, and block-graft copolymers for phase-transition microbubbles as ultrasound contrast agents

**DOI:** 10.3389/fphar.2022.968835

**Published:** 2022-10-13

**Authors:** Jianbo Huang, Hong Wang, Lei Huang, Yuqing Zhou

**Affiliations:** ^1^ Department of Ultrasound, Laboratory of Ultrasound Medicine, West China Hospital, Sichuan University, Chengdu, China; ^2^ College of Polymer Science and Engineering, State Key Laboratory of Polymer Materials Engineering, Sichuan University, Chengdu, China

**Keywords:** phospholipid-mimicking, copolymer, phase-transition microbubbles, ultrasound contrast agent, drug delivery

## Abstract

**Background:** Lipid and polymer microbubbles (MBs) are widely used as ultrasound contrast agents in clinical diagnosis, and possess great potential in ultrasound-mediated therapy due to their drug loading function. However, overcoming the limitations of stability and echo enhancement of MBs are still a considerable challenge.

**Methods:** A series novel block, graft and block-graft copolymers was proposed and prepared in this work, and these copolymers were used as shells to encapsulate perfluoropentane as ultrasound contrast agents. First, block, graft and block-graft copolymers with different topological structures were prepared. Then, these copolymers were prepared into block copolymer phase-transition MBs, graft copolymer phase-transition MBs, and block-graft copolymer phase-transition MBs, respectively. Finally, the dexamethasone was used for drug-loaded phase-transition microbubbles model to explore the potential of theranostic microbubbles.

**Results:** Finally, these three resulting copolymer MBs with average size of 4–5 μm exhibited well enhancement of ultrasound imaging under the influence of different frequencies and mechanical index, and they exhibited a longer contrast-enhanced ultrasound imaging time and higher resistance to mechanical index compared with SonoVue *in vitro* and *in vivo*. *In vitro* drug release results also showed that these copolymer MBs could encapsulate dexamethasone drugs, and the drug release could be enhanced by ultrasonic triggering. These copolymer MBs were therapeutic MBs for targeted triggering drug release.

**Conclusion:** Therefore, the feasibility of block, graft, and block-graft copolymers as ultrasonic contrast agents was verified, and their ultrasonic enhancement performance *in vitro* and *in vivo* was compared. The ultrasound contrast agents developed in this work have excellent development potential in comprehensive diagnosis and treatment.

## 1 Introduction

Ultrasound has been widely used in the diagnosis and treatment of diseases due to the advantages of real-time imaging, portability, safety and combined therapy (Deshpande et al., 2010; Boissenot et al., 2016). Ultrasound contrast agents can enhance the ultrasonic Doppler signal of blood flow and improve the clarity and resolution of ultrasonic images. Contrast-enhanced ultrasound (CEUS) imaging provides a better strategy for ultrasound diagnosis, interventional therapy and targeted drug delivery (Su et al., 2021; Wei et al., 2021; Fang et al., 2022; Sharma et al., 2022). Currently, commercial ultrasound contrast agents consist of gas-encapsulated microbubbles (MBs) are formed by phospholipids or albumin (Quaia, 2007; Díaz-López et al., 2010). However, these MBs have some stability problems, such as coalescence and core dissolution, which result in a short time of gas dissolution in blood circulation under ultrasonic irradiation (Martinez et al., 2010; Mullin et al., 2011; Yang et al., 2013; Huang et al., 2016; Zhang et al., 2016). Therefore, the follow-up research direction of MBs is to prolong the action time and realize the integration of diagnosis and treatment.

Among the new MBs that have been researched, polymers (Cui et al., 2005; Sanna et al., 2011; Park et al., 2012) can form more stable polymer MBs after encapsulating the gas core, which improve their resistance to mechanical pressure changes and have better stability under the action of ultrasound compared with phospholipid MBs. Such polymer MBs can remain intact under low acoustic pressure with almost no volume expansion, and burst only when the pressure exceeded a certain intensity (Bloch et al., 2004). Polymer MBs can also be used as sensitizers for ultrasound image-guided therapy by encapsulating drugs (McEwan et al., 2014; Hasannia et al., 2022), and for multimodal imaging (Ao et al., 2010; Sun et al., 2012; Song et al., 2014; Xu et al., 2015). Comb-like fluorinated blocks add to PEG-PLA polymers enhanced the echogenicity, while the enhancement was lower than that of SonoVue MBs (Houvenagel et al., 2018). Copolymer nanocapsules have several advantages compared to other nanostructures used as drug delivery systems comprising better stability, facile synthesis, prolonged circulation time, and passive/active targeting capability (Petersen et al., 2013). Copolymers with different structures have different effects on molecular weight and surface function (Hasannia et al., 2022). However, a comparative study of different topological structures copolymer as MBs and the effect of these copolymer shells on the ultrasound contrast effect are still lacking. It is very urgent to develop MBs application in targeted ultrasound diagnosis and treatment with higher stability and more prolonged circulatory times *in vivo*.

Therefore, a novel approach was proposed in which we used phospholipid-mimicking block, graft, and block-graft copolymer shells encapsulating perfluoropentane and dexamethasone as drug-loaded MBs. Utilizing the excellent thermoplasticity, biocompatibility and slow degradation rate of polycaprolactone (PCL) (Woodruff and Hutmacher, 2010; Dash and Konkimalla, 2012), the surface of PCL was modified to improve the properties of the material (Rana and Matsuura, 2010). Phosphatidylcholine is the hydrophilic end group of lecithin, and a chemical composition similar to the surface of the cell membrane was obtained by grafting phosphatidylcholine -containing molecules onto the surface of the material. It has good biocompatibility and anti-nonspecific protein adsorption performance (Ishihara et al., 1990; Schlenoff, 2014). In this paper, three topological structures copolymers were prepared, and were then prepared into different drug-loaded phase-transition MBs, such as block copolymer phase-transition MBs (BCPTM), graft copolymer phase-transition MBs (GCPTM) and block-graft copolymer phase-transition MBs (BGCPTM). *In vivo* and *in vitro* results demonstrated that these three copolymers MBs exhibited good enhancement of ultrasound imaging under the influence of different frequencies and mechanical index, and possessed a longer CEUS imaging time and higher resistance to mechanical index compared with SonoVue.

## 2 Materials and methods

### 2.1 Materials

The ε-Caprolactone and 2-hydroxyethyl methacrylate (HEMA) were purchased from Sigma-Aldrich (Shanghai) and were distilled from CaH_2_ before use. 2-Chloro-2-oxo-1,3,2-dioxaphospholane (COP) was purchased from Tokyo Chemical Industry Ltd. (Shanghai). The 2-methacryloyloxyethyl phosphorylcholine (MPC) was synthesized and purified as per Ishihara et al. The 2-bromoisobutyryl bromide, copper (I) bromide (Cu(I)Br; 99.999%), 2,2′-bipyridine (bpy), dexamethasone (Dex), tetrahydrofuran (THF), methanol (MeOH) and all other solvents, reagents with analytical reagent grade were purchased from Shanghai Aladdin Biochemical Technology Co., Ltd.. The 1,2-dipalmitoyl-sn-glycero-3-phosphoethanolamine-N-[methoxy(polyethylene glycol)-5000] (DPPE-mPEG-5000) was purchased from Nanocs Inc. (USA). The perfluoropentane (PFP) and agarose were purchased from J&K Chemical Ltd. (Shanghai). All aqueous solutions were prepared with deionized water. Dialysis membranes (MWCO = 3,500 Da) were obtained from Beijing Solarbio Technology Ltd.

### 2.2 Synthesis of graft copolymer PCL_40_-*g*-PMPC_5×5_

#### 2.2.1 Synthesis and characterization of α-bromocyclohexanone

The α-bromocyclohexanone was synthesized by the slightly modified method of Wang (Wang et al., 2005). The cyclohexanone (31.00 g, 0.3159 mol) and deionized water (200.0 ml) were added to a flask (500 ml) and stirred with a magnetic rotor. Then, bromine (50.61 g, 0.3167 mol) was added dropwise for 5 h, during which the temperature was maintained between 25°C and 30°C by external cooling. After addition was completed, stirring was continued until the reaction mixture was colorless (approximately 1 h). The heavy organic layer was separated from the aqueous layer and dried over anhydrous MgSO_4_. Pure α-bromocyclohexanone (26.7 g, 47% yield) was obtained by distillation.^1^H-NMR (400 MHz, CDCl_3_, ppm): δ 1.65–2.05 (m, 4H, –*CH*_*2*_ *CH*_*2*_–), 2.18–2.36 (m, 2H, CO*CH*_*2*_), 2.93–3.00 (m, 2H, *CH*_*2*_), 4.42–4.44 (t, 1H, *CH*–Br).

Nuclear magnetic resonance hydrogen spectroscopy (^1^H NMR) was measured on a Bruker nuclear magnetic resonance spectrometer (400 MHz). The test solvents were CDCl_3_ and CD_3_OD, and tetramethylsilane was used as the internal standard. The infrared spectrum of the polymer was measured by the KBr tablet pressing method on a Nicolet Magna 560 Fourier transform infrared spectroscopy (FT-IR) instrument. The scanning wavelength range was 500 ∼ 4,000 cm^−1^, and the resolution was 4 cm^−1^. The molecular weight and molecular weight distribution of the polymer were measured by gel permeation chromatography (GPC). The temperature was 40°C, and the flow rate was controlled at 0.6 ml/min. Polystyrene was used as the standard sample. The thermal properties of the polymer were characterized by differential scanning calorimetry (DSC) as described in SI.

#### 2.2.2 Synthesis and characterization of α-bromo-ε-caprolactone

3-Chloroperoxybenzoic acid (36.80 g, 0.1599 mol, 75%) was added to a solution of α-bromocyclohexanone (26.70 g, 0.1508 mol) in CH_2_Cl_2_ (200.0 ml). After stirring at room temperature for 48 h, the reaction flask was placed in a refrigerator for 3 h to precipitate 3-chlorobenzoic acid generated in the reaction. The solution was then filtered and washed with a saturated solution of Na_2_S_2_O_3_ (50.00 ml) 3 times, with a solution of NaHCO_3_ (50.00 ml) 3 times, and finally with deionized water until pH = 7.0. The organic phase was dried with anhydrous MgSO_4_ overnight. After MgSO_4_ was filtered off, the solvent CH_2_Cl_2_ was removed by rotary evaporation. The crude product was dissolved in a mixture of petroleum ether and ethyl acetate (10/3, V/V) and passed through a silica gel column prepared with the same solvent, collecting the second fraction. The solvent was removed by rotary evaporation, and the white solid was dried under vacuum overnight at room temperature. Yield: 20.20 g (69%). ^1^H-NMR (400 MHz, CDCl_3_, ppm): δ 0.75–1.40 (m, 6H, -*CH*_*2*_ *CH*_*2*_ *CH*_*2*_-), 3.4–4.0 (m, 2H, -COO*CH*_*2*_-), 4.4 (m, 1H, -*CH*(Br)-) ppm.

#### 2.2.3 Synthesis of P(BrCL_p_-*co*-CL)_m_

P(BrCL_p_-*co*-CL)_m_ was synthesized by ring-opening polymerizations of αBrCL and εCL (Cao et al., 2015; Yu et al., 2018). In P(BrCL_p_-*co*-CL)_m_, p representes the average number of αBrCL molecules on the copolymer backbone, and m represents the degree of polymerization. There were two kinds of degree of polymerizations for copolymers in this work, i.e., P(BCL_5_-co-CL)_40_ and P(BCL_5_-*co*-CL)_79_. For P(BCL_5_-*co*-CL)_40_, briefly, αBrCL (0.9670 g, 5.010 mmol), εCL (4.000 g, 35.04 mmol), and lauryl alcohol (0.1860 g, 1.000 mmol) were added to a Schlenk flask and stirred with a magnetic rotor. Then, the catalyst stannous octoate (5.200 mg, 0.1 wt%) was added to the former mixture. After a deoxygenation operation, the mixture was reacted at 120°C for 24 h under an N_2_ atmosphere. The crude product was dissolved in CH_2_Cl_2_, and cold methyl alcohol was used to form a precipitate. The final product (P(BrCL_5_-*co*-CL)_40_, 5.118 g, 93%) was dried at 35°C in a vacuum oven for 24 h.

#### 2.2.4 Synthesis of PCL_m_-*g*-PMPC_n×p_

PCL_m_-*g*-PMPC_n×p_ was synthesized by an ARGET (activators regenerated by electron transfer) ATRP method. The m represents the degree of polymerization for P(BrCL-*co*-CL), the n represents the degree of polymerization of the PMPC side chain, and the p represents the average number of PMPC side chains on the copolymer backbone. For PCL_40_-g-PMPC_5×5_, Me_6_TREN (20 μl), CuBr_2_ (8.200 mg), and a mixed solution of THF/MeOH (V/V = 6/6 ml) were added to a Schlenk flask and stirred with a magnetic rotor. Then, MPC (1.117 g), P(BrCL_5_-*co*-CL)_40_ (0.3615 g), and vitamin C (6.500 mg) were added under an N_2_ atmosphere, and the Schlenk flask was closed by a triple valve. After three freeze–pump–thaw cycles, the flask was filled with N_2_, and the mixture was reacted at 35°C for 24 h under an N_2_ atmosphere. The crude solution was dialyzed against deionized water for 72 h. Then, the resulting suspension was freeze-dried, and pure comb copolymer PCL_40_-*g*-PMPC_5×5_ was obtained (99.3%).

### 2.3 Synthesis of block-graft copolymer PCL_34_-*b*-(PBrCL_5_-*g*-PMPC_5×5_)

#### 2.3.1 Synthesis of PCL_34_

Lauryl alcohol (0.82 g, 0.004 mol), ε-CL (10.13 g, 0.089 mol) and Sn(Otc)_2_ (4.05 g, 0.01 mol) were added to the reaction vessel. Remove the O_2_ and residual water. Reaction at 120°C under vacuum for 24 h. The crude product was dissolved in CH2Cl2 and purified by cold methanol precipitation to obtain PCL_34_.

#### 2.3.2 Synthesis of PCL_34_-*b*-PBrCL_5_

PCL_34_ (5.00 g) (obtained in 2.3.1) and αBrCL (0.9670 g, 5.010 mmol) (obtained in 2.2.2) were added to the Schlenk flask and stirred. Then, a catalyst of stannous octanate (5.200 mg, 0.1 wt%) was added to the previous mixture. After deoxidation, the mixture was reacted at 120°C for 24 h in N_2_. The crude product was dissolved in CH_2_Cl_2_, and precipitated with cold methanol. The final product PCL_34_-*b*-PBrCL_5_ was obtained.

#### 2.3.3 Synthesis of PCL_34_-*b*-(PBrCL_5_-*g*-PMPC_5×5_)

The block-graft copolymer of PCL_34_-*b*-(PBrCL_5_-*g*-PMPC_5×5_) was synthesized by the ARGET (electron transfer regeneration activator) ATRP method, where 5 × 5 in PMPC_5×5_ represents the degree of polymerization of the PMPC side chain as 5, and the average value of the PMPC side chain on the main chain is 5. A mixture of Me6TREN (20 μl), CuBr2 (8.200 mg), and THF/MeOH (V/V = 6/6 ml) was added to the Schlenk flask and stirred. Then, MPC (1.117 g), PCL_34_-*b*-PBrCL_5_ (0.3615 g) and vitamin C (6.500 mg) were added under N_2_. Three freezing-vacuuming-melting cycles were carried out, and the mixture was reacted at 35°C in N_2_ for 24 h. The dialysate crude solution was allowed to react for 72 h, then, the suspension was freeze-dried, and the copolymer PCL_34_-*b*-(PBrCL_5_-*g*-PMPC_5×5_) was obtained.

### 2.4 Synthesis of block copolymer PCL_43_-*b*-PMPC_25_

#### 2.4.1 Synthesis of PCL-Br

PCL (7.00 g, 0.002 mol) (obtained in 2.3.1) and triethylamine (0.96 ml, 0.007 mmol) were dissolved in THF. In a N_2_ atmosphere, 2-bromoiso-butyryl bromide (0.85 ml, 0.0069 mmol) was dropped into the solution at −20°C. The reaction was carried out at room temperature for 48–72 h. The quaternary ammonium salt was removed through a column of neutral alumina. A small amount of crude products containing THF were precipitated and vaporized in cold methanol to obtain the white powdered polymer PCL-Br.

#### 2.4.2 Synthesis of PCL_43_-*b*-PMPC_25_

PCL-Br (1.99 g, 0.596 mmol), MPC (6.15 g, 0.021 mol) and Bpy (0.28 g, 1.79 mmol) were dissolved in CH_2_Cl_2_, in N_2_ for 30 min. CuBr (0.13 g, 0.894 mmol) was added to the previous mixture. After 24 h of reaction, purification by neutral alumina column, and further purification with ultra-pure water dialysis. The product PCL_43_-*b*-PMPC_25_ was obtained by freeze-drying.

### 2.5 Preparation of phase-transition MBs

These three copolymers of block copolymer PCL_43_-*b*-PMPC_25_, graft copolymer PCL_40_-*g*-PMPC_5×5_, and block-graft copolymer PCL_34_-*b*-(PBrCL_5_-*g*-PMPC_5×5_) were obtained by the above method. The block copolymer (PCL_43_-*b*-PMPC_25_) nanodroplets were prepared by the emulsion/nanoprecipitation technique(Zhu et al., 2019). First, 3.0 mg of PCL_40_-*g*-PMPC_5×5_ and 1.0 mg of DPPE-mPEG-5000 were dissolved in 850.0 μl of THF/MeOH mixed solution (2:1 v/v). Then, 2.0 ml of phosphate buffer saline (PBS, 0.01 M, pH 7.4) and 150.0 μl (2% v/v) of PFP liquid were added into the mixed solution. After that, the solution was ultrasonic emulsification with a probe by an ultrasonic processor (SCIENTZ–IID) at 35 Watt for 5 min in an ice bath environment. The resultant nanodroplets were centrifuged at 5000 *×* g for 5 min to remove residual THF, MeOH and PFP. This nanodroplets were then dialyzed against PBS for 24 h at 4°C to remove residual organic solvents and unloaded PFP, and then stored at 4°C.

**SCHEME 1:**
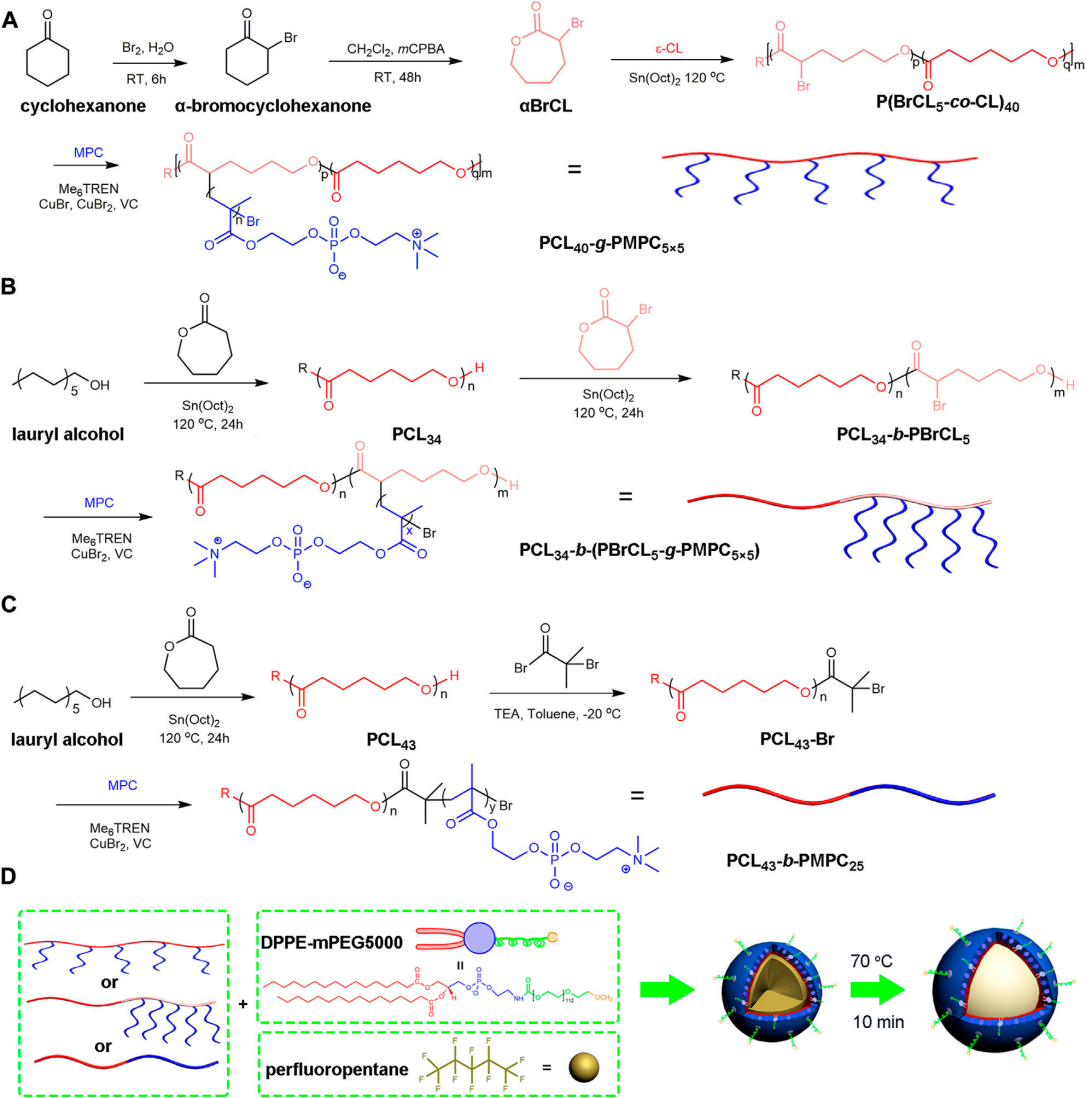
Schematic illustration of the preparation and echogenic properties of the BCPTM, GCPTM and BGCPTM. **(A)** Synthesis of PCL_40_-*g*-PMPC_5×5._ **(B)** Synthesis of PCL_34_-*b*-(PBrCL_5_-*g*-PMPC_5×5_) **(C)** Synthesis of PCL_43_-b-PMPC_25_. **(D)** Synthesis of BCPTM, GCPTM and BGCPTM.

To obtain the activation block copolymer (PCL_43_-*b*-PMPC_25_) phase-transition MBs (BCPTM), the resultant copolymer NDs were incubated for 10 min at 70°C in a water bath shaker. Block-graft copolymer (PCL_34_-*b*-(PBrCL_5_-*g*-PMPC_5×5_)) phase-transition MBs (BGCPTM) and graft copolymer (PCL_40_-*g*-PMPC_5×5_) phase-transition MBs (GCPTM) were obtained in the same experimental steps and stored at 4 °C. A schematic illustration of the preparation of the PFP-entrapped MBs is shown in Scheme 1.

### 2.6 Size distribution and zeta potential

The hydrodynamic diameter (*d*_H_) and zeta potential of the nanodroplets and activation MBs were measured by dynamic light scattering (DLS, Zetasizer Nano ZS instrument, Malvern, France). 10 μl of suspensions were diluted in 2 ml water. Measurements were performed at 20°C, at an angle of 173° to avoid multiple scattering. Zeta potential measurements were carried out in PBS at 25°C with the same instrument. Measurements were performed in triplicate. We measured the concentration of these activation MBs using a 20 μm small pore tube of BECKMAN COULTER Multisizer 4, and then uniformly diluted them to 1.0 × 10^9^ bubbles/mL in PBS for subsequent experiments.

### 2.7 *In vitro* cytotoxicity evaluation

Human umbilical vein endothelial cells (HUVECs) were purchased from ATCC and cultured according to the supplier’s instructions. The cells were seeded into a 96-well plate (1 × 10^4^ cells/well) and incubated overnight. Subsequently, 100 µl samples of BCPTM, GCPTM and BGCPTM were added into each group separately. Three duplicate wells were set in each group, and the MBs were co-incubated with for 8 and 24 h separately. The cytotoxicity of these MBs was examined by a cell counting kit assay (CCK-8, Abcam, Shanghai, China). CCK-8 solution (10% (v/v)) was added in and incubated for 1 h. The optical density was measured at 450 nm by a Varioskan Flash microplate reader (Synergy Mx, BioTek Instruments, VT, United States), and the cell viability was calculated as the described method (Qin et al., 2021a).

In addition, calcein-AM/Propidium iodide (PI) was used to assess the cell state after MBs treatment. After The cells were co-incubated with MBs for 8 and 24 h separately, calcein-AM (1 μM) was added in and incubated for 15 min PI (20 μM) was added as a dead cell stain. The fluorescence images of the treated cells were collected by an inverted fluorescence microscope (AX10 imager A2/AX10 cam HRC, Carl Zeiss, Germany).

A 2% of sheep red blood cells (accretion) was used to verify the hemolysis of these three MBs. 500 μl MBs suspensions at concentrations of 10, 25, 50, 100, 200, and 400 μl/ml were added to erythrocyte suspensions (500 µl). Equal volume of PBS and double-distilled water was used as the control group. All mixtures were incubated at 37°C for 2 h and centrifuged at 1,000 × g for 10 min. 100 μl of supernatant was placed on a 96-well plate, and the optical density of each group was measured at 570 nm using the Varioskan Flash microplate reader.

### 2.8 *In vitro* CEUS imaging

*In vitro* CEUS imaging was performed in an agar gel hole (1.5 W/V %) using an ultrasound system (ACUSON Antares P.E., SIEMENS Healthineers, Germany) with a probe VFX13-5 (SIEMENS, Germany). A glass stirring rod was inserted into the agarose solution to cool, and then pulled out to form a sample loading hole of pproximately 300 µl volume. 200 µl of samples was injected into the hole. The CEUS images were acquired in cadence contrast agent imaging mode at different ultrasonic parameters, such as a frequency range of 4.00–10.00 MHz, mechanical index (MI) range of 0.05–0.67, concentration range of 1-40X, and time range of 0–20 min. Then the mean gray intensity (MGI) of the region of interest of all CEUS images was processed and analysed using MATLAB 2016b (MathWorks Inc., United States).

### 2.9 *In vivo* CEUS imaging

In order to verify the ultrasound enhancement of BCPTM, GCPTM and BGCPTM as MBs *in vivo*, the CEUS imaging was performed on the liver of 8 week-old healthy New Zealand rabbits (CHENGDU DOSSY EXPERIMENTAL ANIMALS CO., LTD., China). Animal experiments were all carried out according to the guidelines of animal care and use of the Animal Ethics Committee in West China Hospital, Sichuan University, Chengdu, China. The rabbits (weighing of 2.0–2.5 kg) were anaesthetized with 2% isoflurane, and then injected with 200 μl/kg weight of PBS, BCPTM, GCPTM and BGCPTM (∼1.0 × 10^9^ bubbles/ml) or SonoVue (∼1.1 × 10^8^ bubbles/ml) through the marginal auricular vein, 5 rabbits in each group. After that, the rabbit’s liver was monitored and imaged by an Acuson Antares ultrasound imaging system (SIEMENS) in cadence contrast agent imaging mode at a frequency of 5.71 MHz and MI value of 0.20.

### 2.10 *In vitro* drug release

These MBs of BCPTM, GCPTM and BGCPTM were used to measure the amount of drug released separately. During the preparation of the copolymer nanodroplets, 3.0 mg Dex was added to the mixed solution before ultrasonic emulsification, and then activated into phase- transition MBs. Then, 0.5 ml of each UCA was added in dialysis bag (Mw 3500) and immersed in 40 ml of PBS dialysis buffer in a shaking water bath at 37°C. The US- triggered groups were subjected to ultrasound irradiation with 1 MHz (3 W/cm^2^, 20% duty cycle, 3 min at 1 h). 2 ml of dialysis buffer was collected for measurement, and 2 ml of new PBS buffer was added at each measurement time. The Dex release amount of the samples collected at each measurement time was determined and calculated based on the standard calibration line *via* UV absorbance at 240 nm using a UV-Vis spectrophotometer. The Dex release curves of these MBs of BCPTM, GCPTM and BGCPTM were calculated as in previous studies (Zhu et al., 2019).

### 2.11 Statistical analysis

Data were analysed with SPSS version 22.0 software (USA). Mean ± standard deviation (SD) was used to describe all data, and the means between 2 groups were compared using one-way ANOVA, followed by the Kruskal-Wallis test. Differences were considered significant at *p* < 0.05. Measurements of each sample were carried out three times.

## 3 Results

### 3.1 Characterization of these three copolymers

**FIGURE 1:**
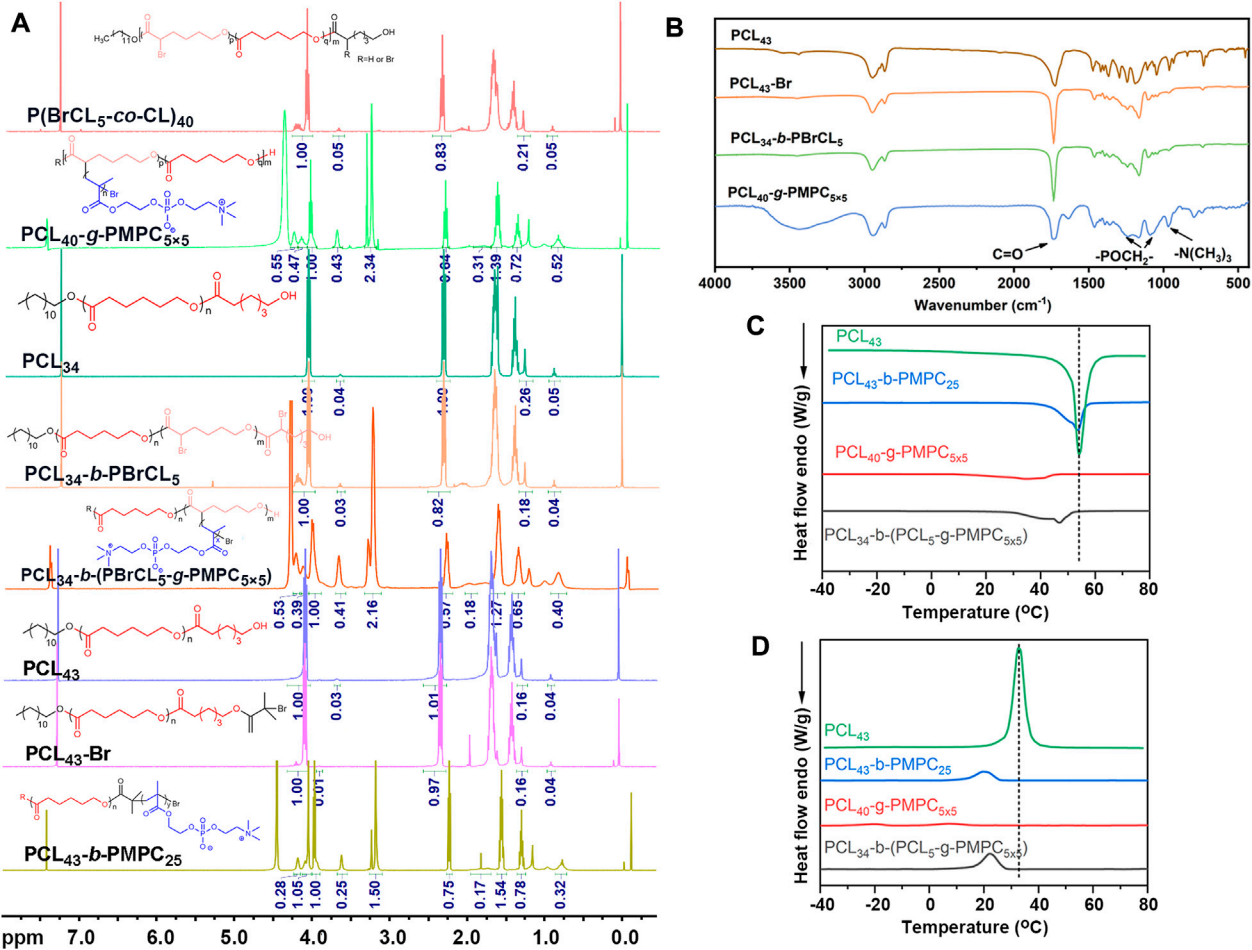
**(A)** 400 M ^1^H NMR spectra of different copolymers in CDCl_3_. **(B)** FTIR spectra of different copolymers. **(C)** DSC curve of different copolymers for heating. **(D)** DSC curve of different copolymers for cooling.

The NMR spectrum of the obtained copolymer was shown in Figure 1A. Segment characteristic peaks of PCL (δ = 4.1 ppm; δ = 2.3 ppm; δ = 1.7 ppm; δ = 1.4 ppm) and PMPC (δ = 4.32 ppm; δ = 4.21 ppm; δ = 3.74 ppm; δ = 3.29 ppm; δ = 1.83 ppm; δ = 0.89 ppm) appeared in the nuclear magnetic spectrum (Tu et al., 2011). At the same time, the total polymerization degree of PMPC could be calculated by the integral area ratio of the R peak to the C peak. The NMR results showed that the PCL-PMPC polymers were successfully synthesized (Figure 1A). PCL and PCL-PMPC copolymer of theoretical content and elemental analysis were shown in Supplementary Table S2. The 400 M ^1^H NMR spectrum of P (BCL-co-CL) (Supplementary Figure S1). GPC curves of PCL_34_, PCL_34_-*b*-PBCL_5_ and PCL_43_-Br (Supplementary Figure S2).

The structure of the polymer was further characterized by infrared spectroscopy (Figure 1B). The peak at 1728 cm^−1^ is the stretching vibration peak of C = O, the continuous methylene absorption peak at 2910 cm^−1^ and the C-Br vibration peak at 580 cm^−1^. Compared with P (BCL_5_-co-CL)_40_, PCL_40_-*g*-PMPC_5×5_ showed characteristic absorption peaks related to the structure of phosphatidylcholine (1,090 cm^−1^ and 1,230 cm^−1^, - poch_2_ -; 970 cm^−1^, N^+^ (CH_3_) _3_). Therefore, the infrared results showed that the PCL_40_-*g*-PMPC_5×5_ polymer was successfully synthesized.

The thermal properties of the synthesized polymer were tested by DSC (Figures 1C,D; Supplementary Table S1). The PCL homopolymer had the highest crystallization temperature, melting temperature and crystallinity. Compared with PCL, three different structures of PCL-PMPC copolymers, with the introduction of amorphous PMPC segments, the regular arrangement of segments was blocked during the crystallization of PCL, resulting in the reduction of various data.

### 3.2 Characterization of BCPTM, GCPTM and BGCPTM

**FIGURE 2:**
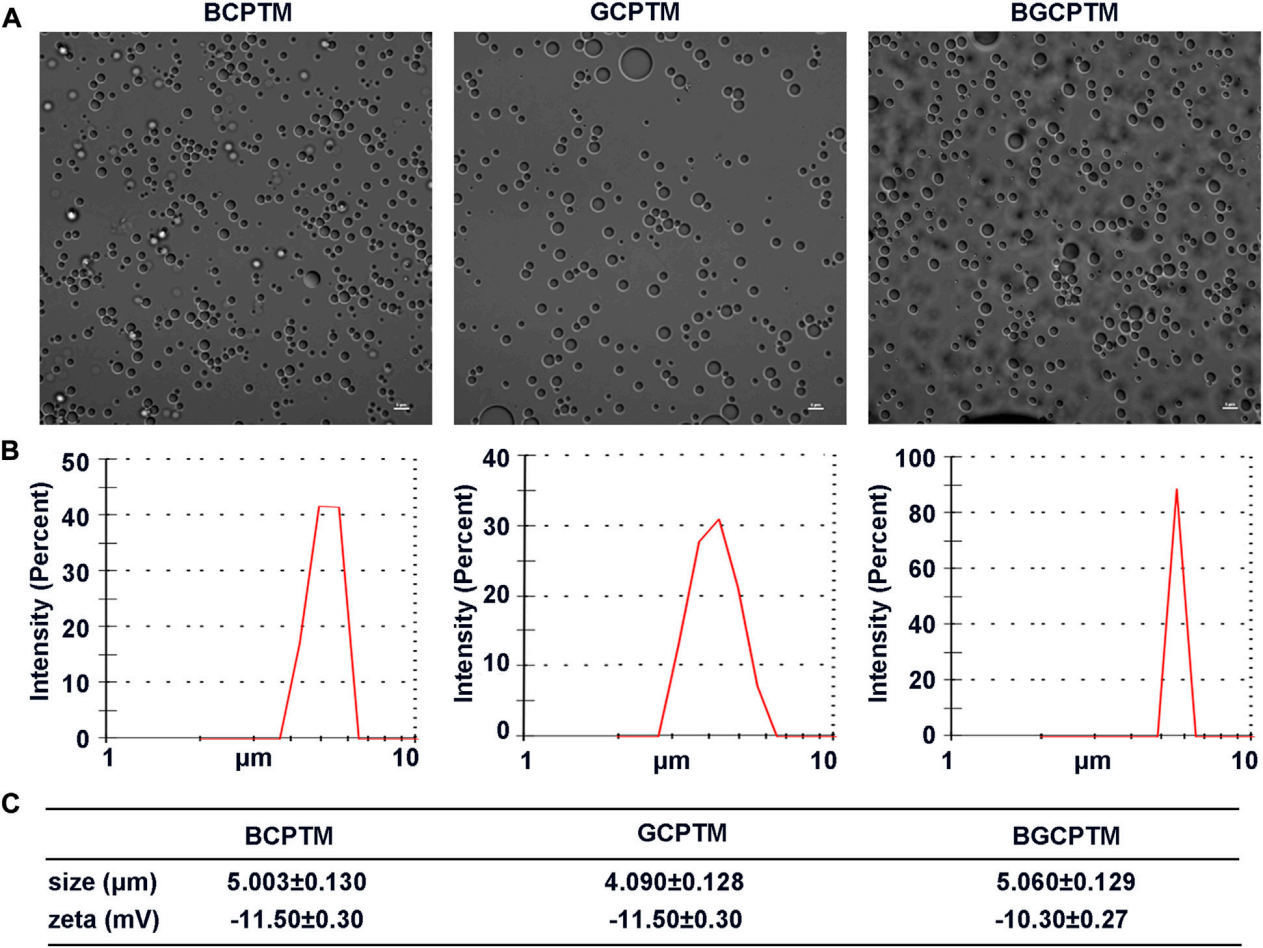
Characterization of BCPTM, GCPTM and BGCPTM. **(A)** Optical microscopy images. **(B)** The size distribution, determined by the DLS. **(C)** The particle sizes and Zeta potentials of these fabricated BCPTM, GCPTM and BGCPTM.

Due to the good compatibility of copolymer and PFP, hydrophobic PFP was successfully encapsulated by copolymer. These three kinds of synthesized MBs were spherical, relatively small and had uniform particle sizes in the optical microscopy images (Figure 2A). The DLS results indicated that the three MBs all had a narrow size distribution (Figure 2B). The particle size of BCPTM was 5.003 ± 0.130 μm. The particle size of GCPTM was 4.090 ± 0.128 μm. The particle size of BGCPTM was 5.060 ± 0.129 μm. The zeta potentials of these fabricated MBs were −11.50 ± 0.30 mV, −11.50 ± 0.30 mV, and −10.30 ± 0.27 mV, respectively (Figure 2C).

### 3.3 *In vitro* cytotoxicity evaluation

**FIGURE 3:**
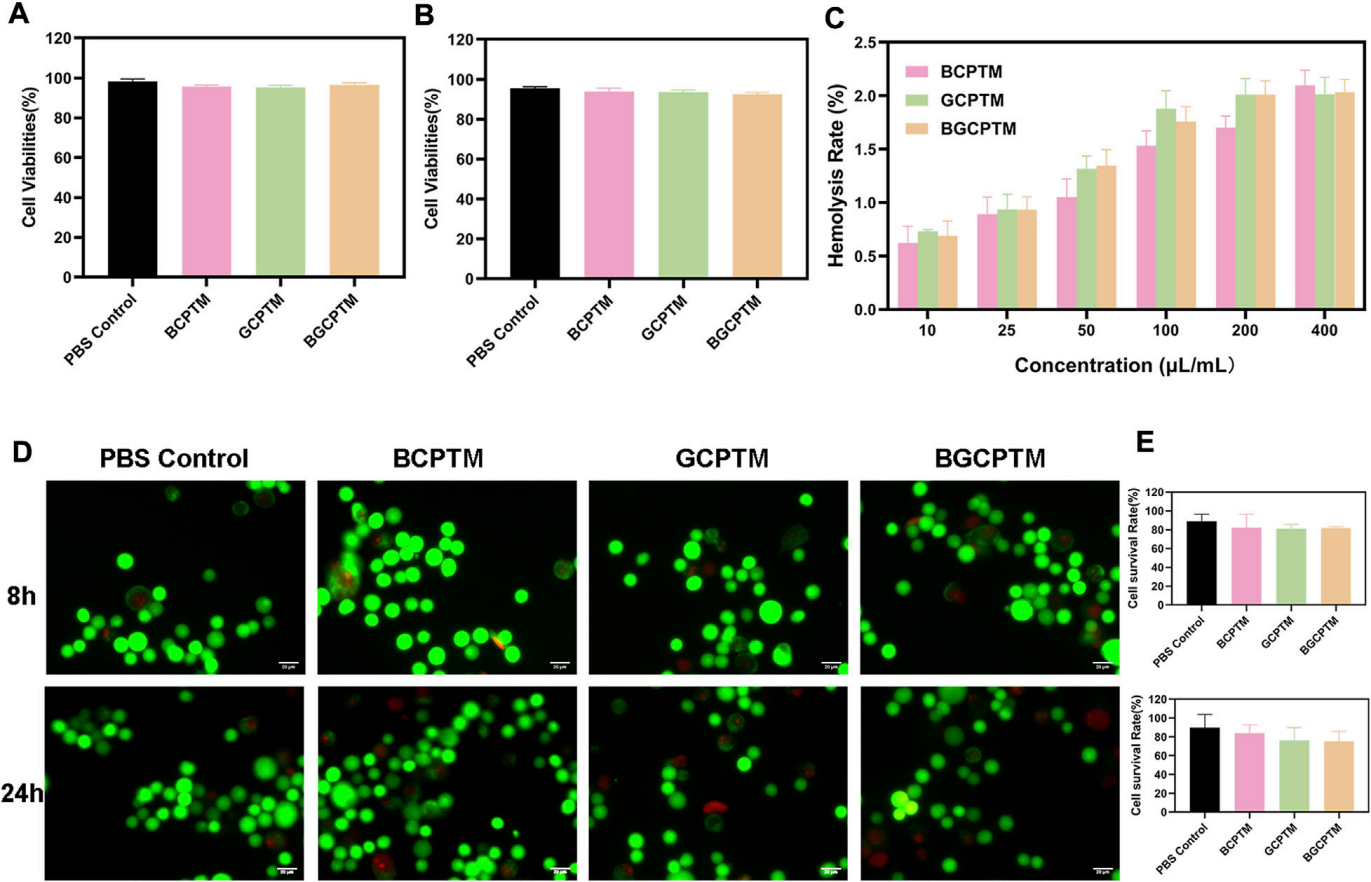
*In vitro* cytotoxicity evaluation and erythrocytes hemolysis. **(A)** Cell viabilities of BCPTM, GCPTM and BGCPTM after co-incubated 8 h (*n* = 3). **(B)** Cell viabilities of BCPTM, GCPTM and BGCPTM after co-incubated 24 h. **(C)** Erythrocytes hemolysis with different concentration. **(D)** Fluorescence images of HUVECs cells co-stained by calcein AM/PI after treated with different preparations. Live and dead cells displayed green and red signals, separately. Scale bar: 20 μm. **(E)** Quantitative analysis of the cell survival rates in Panle **(D)**.

The BCPTM, GCPTM and BGCPTM groups showed no significant difference in the hemolysis ratio at any concentration and compared with the PBS group there was no significantly inhibit cell proliferation (Figure 3). This indicated that BCPTM, GCPTM and BGCPTM had good biocompatibility and caused negligible cytotoxicity when used separately. The fluorescence images of HUVECs cells costained with calcein-AM/PI also showed no differences when being treated with different preparations at a concentration of 10 μl/ml.

### 3.4 Results of *in vitro* CEUS imaging

**FIGURE 4:**
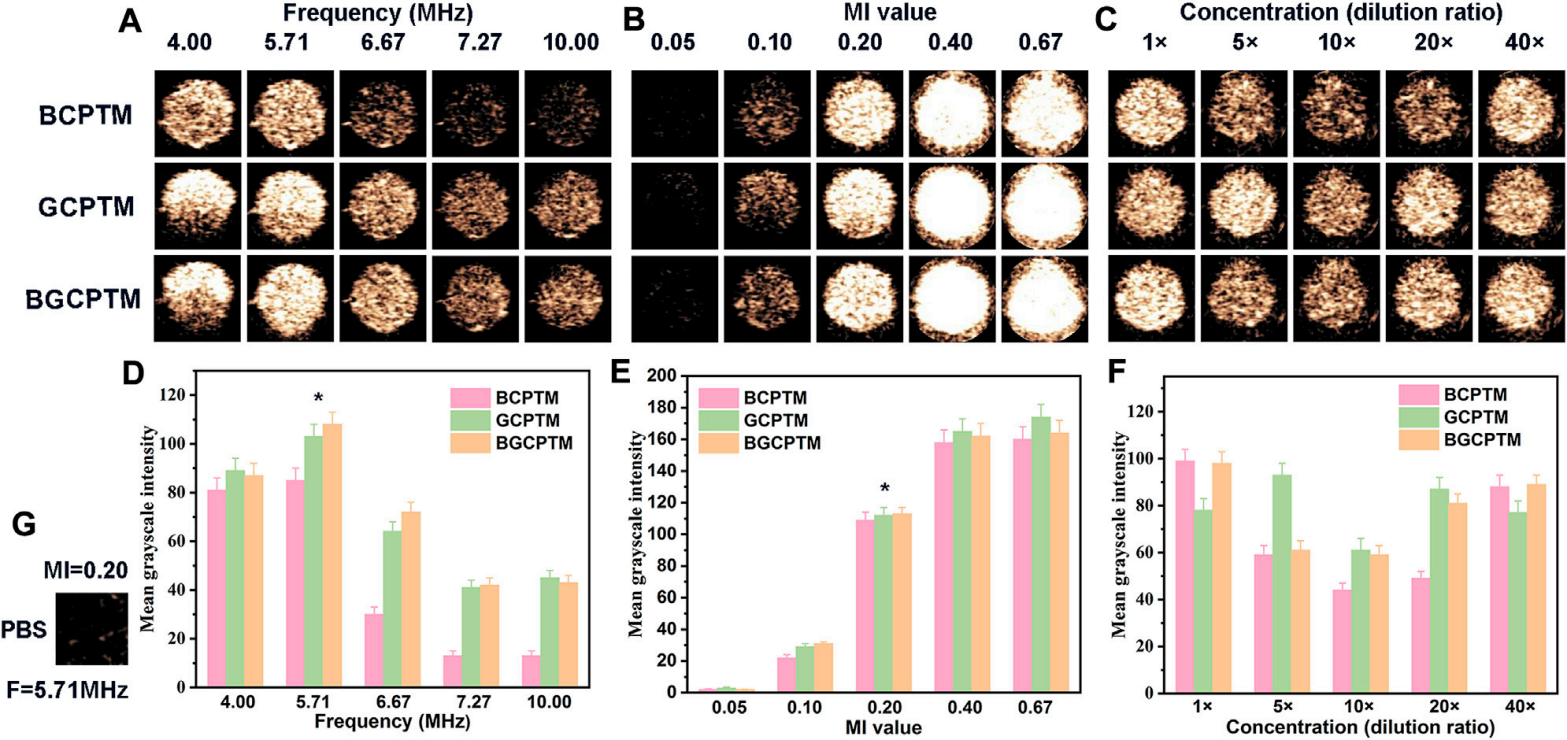
*In Vitro* Ultrasound Imaging. **(A)** CEUS images of BCPTM, GCPTM and BGCPTM under different frequencies *in vitro*. **(B)** CEUS images of BCPTM, GCPTM and BGCPTM under different MI. **(C)** CEUS images of BCPTM, GCPTM and BGCPTM under different concentration. **(D)** MGI of CEUS images under different frequencies. **(E)** MGI of CEUS images under different MI. **(F)** MGI of CEUS images under different concentration. **(G)** As control, the CEUS image of PBS was obtained at 5.71 MHz frequency and 0.20 MI, and no enhancement was detected in this image.

The MGI of the images representing the echo intensity were different under different ultrasonic frequencies (Figures 4A,D). Generally, the echo intensity of the three MBs groups reached the strongest at 5.71 MHz. The MGI values of the images were reduced to varying degrees when the frequency was lower (4 MHz) or higher than 5.71 MHz (6.67 MHz, 7.27 MHz, 10.00 MHz). The MGI values of these three kinds of MBs at 5.71 MHz were significantly different from those at other frequencies. To evaluate the performance of these kinds of MBs under different mechanical indices, the results showed that the MGI of the image increases with increasing MI (Figures 4B,E). When the MI increases from 0.05 to 0.10, the MGI of the images increases slightly. The MGI under the MI of 0.2 obtained by these three MBs was significantly different from that obtained under other MI values. However, when the MI exceeded 0.40, the rising trend of MGI was no longer evident. In addition, the MBs rapidly burst under the action of high-energy ultrasound, which cannot continuously enhance CEUS imaging. Figures 4C,F shows that at different concentrations from 1× to 40× (the concentration of 1× was 1.02 × 10^9^/ml), there was no stable change trend of the MGI in each MBs group, which suggested that the variation in MBs concentration in a particular range might have little impact on imaging. Based on the above research results, we kept the ultrasonic condition of the frequency of 5.71 MHz and an MI of 0.20 in the subsequent imaging.

**FIGURE 5:**
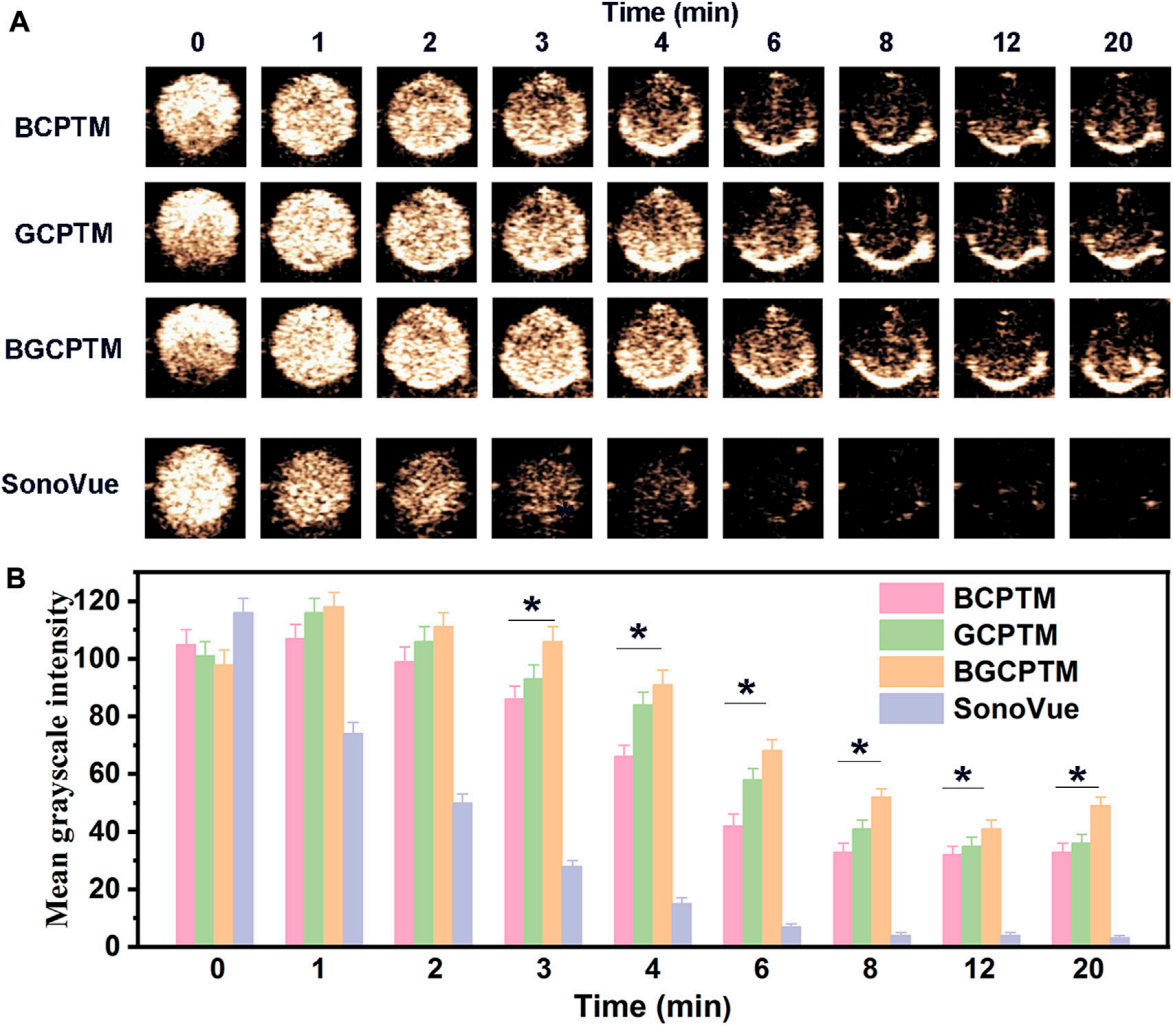
**(A)** Continuous CEUS images for 20 min of BCPTM, GCPTM, BGCPTM and SonoVue *in vitro*. **(B)** MGI of the 20 min CEUS images of BCPTM, GCPTM, BGCPTM and SonoVue *in vitro*.

**FIGURE 6:**
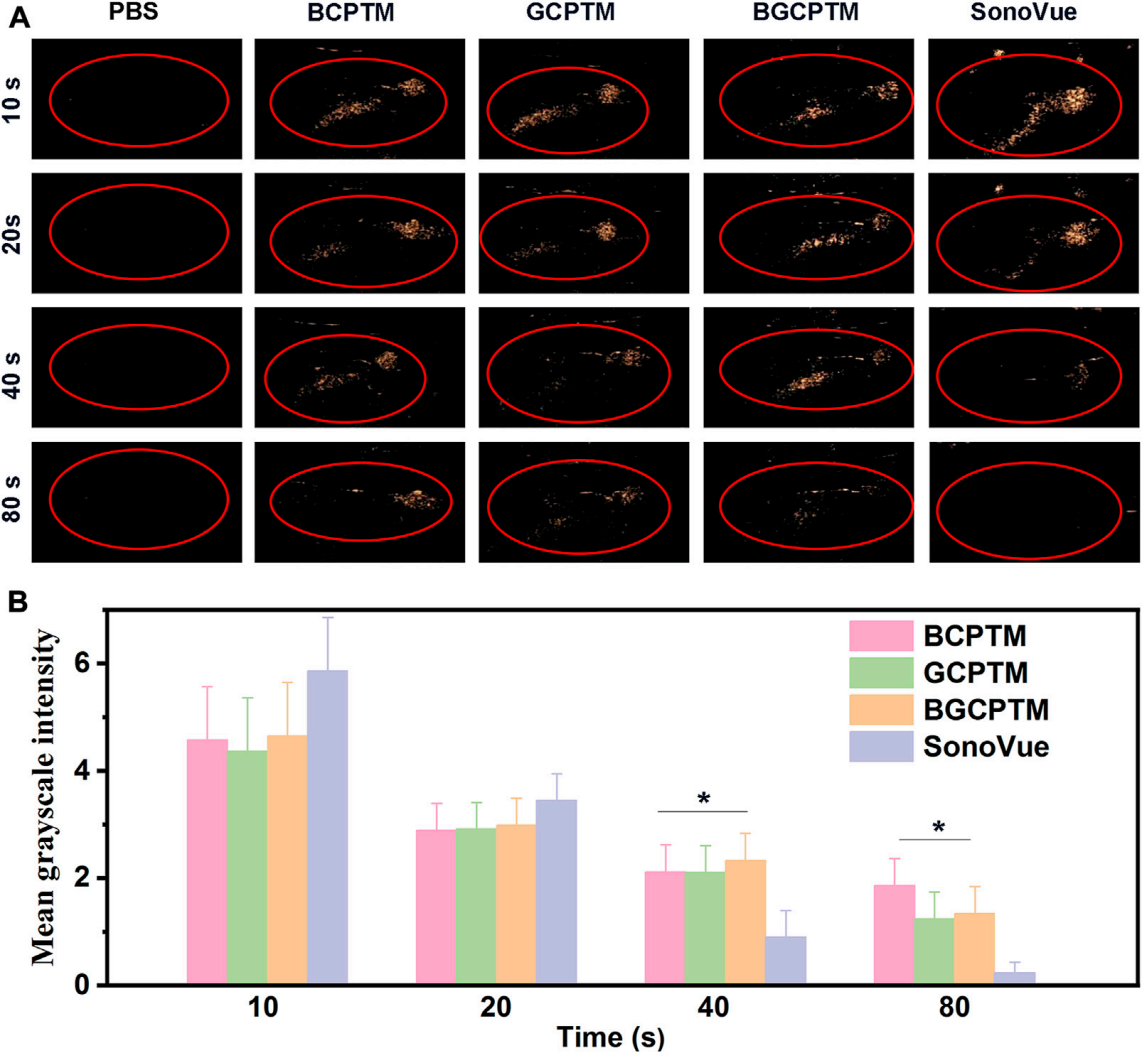
CEUS images of BCPTM, GCPTM, BGCPTM and SonoVue in rabbits liver (*n* = 3). **(A)** Continuous CEUS images for 90 s *in vivo*. **(B)** MGI of the CEUS images.

In studying the stability of BCPTM, GCPTM and BGCPTM, we added SonoVue for comparison (Figure 5). For the echo intensity reflected by the MGI, the imaging contrast of the three MBs and SonoVue was excellent initially. Still, the MGI of the SonoVue group decreased rapidly from 1 min, and it was challenging to observe an obvious enhancement in the SonoVue group after 6 min. The stability of the three MBs was more robust than that of SonoVue in terms of the rate of MGI decreasing with time. Figures 5B, 6B showed the MGI values difference between MBs and SonoVue group at different time points, and then compared with MGI values difference between MBs and SonoVue group at the initial time point. After 3 min the MGI of SonoVue group decreased significantly compared to the other groups. Among them, the BGCPTM group showed the best stability, and even the echo intensity observed at 6 min in these two groups was still stronger than that of the SonoVue group at 2 min. At the end of observation (20 min), no noticeable enhancement could be detected in the SonoVue group, while we could still see a specific degree of enhancement in the BCPTM, GCPTM and BGCPTM groups.

### 3.5 *In vivo* evaluation of CEUS imaging

The *in vivo* CEUS imaging performance was shown in Figure 6. PBS solution was injected as the control group, and the liver region showed no enhancement in the acquired images. Compared with the PBS control, the BCPTM, GCPTM, BGCPTM and SonoVue groups showed noticeable enhancement, indicating that the MBs successfully passed through the pulmonary capillaries and reached the liver vascular system. Specifically, the BCPTM, GCPTM and BGCPTM groups initially showed stronger echo intensity than the SonoVue group, and the contrast of the BGCPTM group was the best among the three MBs. At 40 s, minor enhancement could be observed in the SonoVue group, while hepatic vein structure could still be identified in the BCPTM, GCPTM and BGCPTM groups, the MGI of SonoVue group was significantly lower than the other groups. After 40 s, the echo intensity of each group decreased, and at 90 s, no significant enhancement was observed in the liver structure of the three groups.

### 3.6 Drug encapsulation and release

**FIGURE 7:**
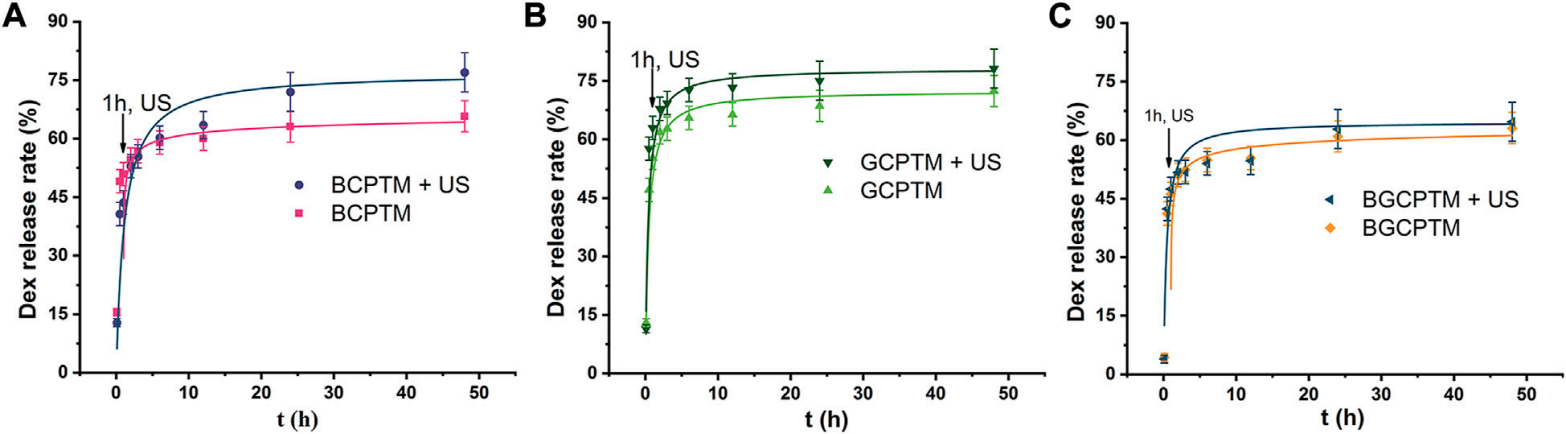
*In vitro* Dex release curve. **(A)** BCPTM, GCPTM and BGCPTM with and without ultrasound irradiation (1 MHz, 3 W/cm^2^, 20% duty cycle, 3 min at 1 h) at 37°C (*n* = 3) for 48 h. **(B)** GCPTM with and without ultrasound irradiation. **(C)** BGCPTM with and without ultrasound irradiation

The *in vitro* Dex release curves of the BCPTM, GCPTM and BGCPTM groups with and without ultrasound irradiation (1 MHz, intensity of 3 W/cm^2^, 20% duty cycle, 3 min at 1 h), where the Dex release rate showed a rapid release (Figure 7). The cumulative amount of drug release ranged from 43.6 ± 3.1% to 63.0 ± 4.3% in the initial 1 h. From 1 h (the time point of ultrasound irradiation) to 24 h, the cumulative release rate of the BCPTM + US group was much higher than that without ultrasound irradiation, and the total amount of drug release in the US- triggered group was 76.99 ± 5.4% (Figure 7A). There was no apparent difference between the BGCPTM and BGCPTM + US groups in drug-release behavior (Figure 7C). However, all groups showed a small amount of slow sustained release from 24 to 48 h.

## 4 Discussion

In recent years, several biocompatible phase-transition nanodroplets composed of organic and/or inorganic materials have been developed for drug delivery systems and photoacoustic tomography (Awad et al., 2021; Heer et al., 2021; Zhao et al., 2022). However, few studies have discussed the feasibility of block, graft and block-graft copolymers used to prepare phase-transition copolymer nanodroplets as MBs, and compared their ultrasonic enhancement performance *in vitro* and *in vivo*.

Generally, the synthesis of PMPC copolymers with ATRP is carried out in polar solvents with tert butyl bromide as the initiator and CuBr/bpy as the catalytic system (Ma et al., 2003). Since more CuBr is used as the catalyst in the experiment, and Cu^2+^ generated after the reaction is difficult to remove in the polymer, we adopted the arget ATRP reaction, in which only a small amount of CuBr (usually ppm level) is required (Matyjaszewski et al., 2007). On the other hand, bromine as the initiation site in the P (BCL co CL) copolymer is connected with a secondary carbon. It also has a large steric hindrance at the position of the main chain, resulting in a significant reduction in its initiation activity (Tang and Matyjaszewski, 2007). Therefore, we used highly active me6tren as a ligand to replace bpy. In conclusion, the PCL-g-PMPC comb copolymer was synthesized by an arget ATRP reaction with P (BCL-co-CL) as a macromolecular initiator and CuBr2/me6tren/VC as a catalytic system.

The boiling temperature of PFP is 29°C (Lattin et al., 2012). Using PFP as the core of nanodroplets may be limited if it is unstable in physiological temperature. The particle diameter of these prepared three phase-transition copolymer nanodroplets remained stable over 24 h at 37°C (Supplementary Figure S3, Supporting Information). Therefore, the results indicated that these copolymer nanodroplets remain relatively stable at body temperature. It can be seen that the Laplace pressure of the copolymers shell significantly improves the phase-transition temperature of PFP (Kee and Teo, 2019). The surface tension on the nanodroplets interface along with the ability to superheat the liquid helps the PFP stay liquid at body temperature. According to the Laplace pressure, encapsulating the droplets in the copolymer shells and the small radius of the droplets are sufficient to stabilize the PFP above its normal boiling point (Zhang et al., 2017). Therefore, the results strongly suggest that the nanodroplets had appropriate size with diameters of approximately 200 nm can passively accumulate around tumor tissues due to the enhanced permeability and retention (EPR) effect (Nyankima et al., 2018) and stability suitable for additional *in vivo* theranostic applications.

These PFP nanodroplets were subjected to a water baths at different temperatures of 60, 65, 70, 75, and 80°C for 10 min and analyzed by CEUS images for phase-transition (Supplementary Figure S4). The results showed that at 60°C, the MGI value of CEUS image was low, resulting in a small amount of thermal MBs. At about 70°C, the MGI value of CEUS image increases, and the echo is significantly enhanced. Finally, at higher temperatures (∼80°C), MGI decreases and MBs begins to rupture and disappear. It can be seen that the copolymer shell greatly increases the phase-transition temperature of PFP core, triggering the phase-transition of PFP at 70°C, and generating a large number of MBs. It has been reported that the boiling point of PFP droplet is related to the boiling point of PFP core, droplet size, shell composition and bulk mode stiffness. The larger nanodroplets particle size, the lower the threshold of phase-transition temperature (Kripfgans et al., 2004). The smaller the diameter of the droplet, the greater the Laplacian pressure, the less likely to phase into MBs (Barber and Cady, 1956; Rapoport et al., 2009; Sheeran et al., 2011; Zhou, 2015; Mountford and Borden, 2016). Recently, studies have shown that the temperature range of PFP droplet is 64 ∼ 76°C (Vidallon et al., 2022). Therefore, nanodroplets could not phase into MB until it reaches the minimum threshold sound pressure or the temperature is above its boiling point (Kee and Teo, 2019). Therefore, the CEUS images and quantitative analysis signified that the occurrence of phase-transition and the intensity of the echogenicity signal occurred in a temperature-dependent manner. Preparation of MBs using the phase-transition nanodroplets for enhanced ultrasound imaging is a common method for real-time ultrasound imaging (Choi et al., 2019; Choi et al., 2020; Qin et al., 2021b). The MBs of BCPTM, GCPTM and BGCPTM designed in this paper can effectively be activated from the phase-transition copolymer nanodroplets into MBs, realizing ultrasound monitoring imaging. Among them, the particle size uniformity of BCPTM MBs was slightly worse than that of others. The particle size of BGCPTM was the largest at 5.060 ± 0.129 μm, and that of GCPTM was the smallest (4.090 ± 0.128 μm). Not only that, compared with the SonoVue, these three MBs have longer CEUS imaging times (Figures 4, 5), which might be related to the high chemical stability, structural perfection and uniform of these phase-transition copolymer MBs. Within a certain range of concentration, increasing its concentration does not enhance the imaging effect of CEUS, which may be related to the nonlinear vibration of MBs (Pang et al., 2018). High concentration of MBs can produce stronger ultrasonic enhancement effect, but inertial cavitation vibration will lead to the collapse of MBs and reduce the concentration of MBs (Duco et al., 2016). This may be due to the rapid rupture of MBs caused by high frequency, which could lead to the rapid decline of contrast enhancement effect. In addition, MBs cannot form resonance effectively under the action of low frequency ultrasound, which also reduced the MGI of the images (Gorce et al., 2000). Furthermore, these phase-transition copolymer nanodroplets can also be activated by HIFU or loaded with some photosensitizers to achieve NIR activation.

The *in vitro* cytotoxicity evaluation results of BCPTM, GCPTM and BGCPTM indicated that the composition and topological structure of the materials had little effect on cytotoxicity and erythrocyte hemolysis. This is due to the good biocompatibility and biodegradability of phase-transition copolymer nanodroplets functionalized with molecules increases their circulation time (Yang et al., 2016). From the Dex release curve, we found that the Dex release rate of the BCPTM, GCPTM and BGCPTM groups was between 60% and 75% (Figure 7). Ultrasound irradiation can increase the drug release rate of each group, suggesting that these MBs have the potential to carry anti-tumor drugs and other drugs, which can increase the concentration of local drugs while CEUS can realize the triggering release of special targets of various diseases and enhance the therapeutic effect of local lesions (Sharma et al., 2022).

## 5 Conclusion

In conclusion, phospholipid-mimicking block, graft, and block-graft copolymers were successfully constructed, and were then used for phase-transition MBs as MBs in this study. These fabricated amphiphilic biodegradable BCPTM, GCPTM and BGCPTM as MBs were prepared to explore the possibility of overcoming the limitations of short ultrasound enhancement time of Sonovue. *In vitro* and *in vivo* results of these MBs demonstrated excellent enhancement in CEUS images. The BCPTM, GCPTM and BGCPTM groups showed significant advantages in CEUS images compared with SonoVue, such as better stability at high frequency and high mechanical index, and still had specific contrast-enhanced effect *in vitro* for 20 min, providing a longer imaging time for CEUS. However, the CEUS effects of BCPTM, GCPTM and BGCPTM did not show significant differences due to their different structures and compositions *in vitro*. The CEUS imaging time of BCPTM, GCPTM and BGCPTM in the liver of New Zealand rabbits was longer than that of SonoVue, while there was no significant difference in the ultrasound effects between the three different topological MBs. Moreover, based on the drug loading ability and the enhancement of drug release rate by ultrasound, these three MBs may be used for integrated diagnosis and treatment. A comparative study of these three MBs laid a foundation for the further application of multifunctional HIFU therapy and photoacoustic imaging agents.

## Data Availability

The original contributions presented in the study are included in the article/Supplementary Material, further inquiries can be directed to the corresponding author.

## References

[B1] Ao M. Wang Z. Ran H. Guo D. Yu J. Li A. (2010). Gd-DTPA-loaded PLGA microbubbles as both ultrasound contrast agent and MRI contrast agent—a feasibility research. J. Biomed. Mat. Res. B Appl. Biomater. 93B (2), 551–556. 10.1002/jbm.b.31614 20225249

[B2] Awad N. S. Paul V. AlSawaftah N. M. ter Haar G. Allen T. M. Pitt W. G. (2021). Ultrasound-Responsive nanocarriers in cancer treatment: A review. ACS Pharmacol. Transl. Sci. 4 (2), 589–612. 10.1021/acsptsci.0c00212 33860189 PMC8033618

[B3] Barber E. J. Cady G. H. (1956). Vapor pressures of perfluoropentanes. J. Phys. Chem. 60 (4), 504–505. 10.1021/j150538a030

[B4] Bloch S. H. Wan M. Dayton P. A. Ferrara K. W. (2004). Optical observation of lipid- and polymer-shelled ultrasound microbubble contrast agents. Appl. Phys. Lett. 84 (4), 631–633. 10.1063/1.1643544

[B5] Boissenot T. Bordat A. Fattal E. Tsapis N. (2016). Ultrasound-triggered drug delivery for cancer treatment using drug delivery systems: From theoretical considerations to practical applications. J. Control. Release 241, 144–163. 10.1016/j.jconrel.2016.09.026 27667179

[B6] Cao J. Cheng F. Cao H. Lu A. Cai M. Chen Y. (2015). Framework effect of amphiphilic polyesters on their molecular movement and protein adsorption-resistance properties. Colloids Surf. B Biointerfaces 125, 213–221. 10.1016/j.colsurfb.2014.11.040 25499227

[B7] Choi H. Choi W. Kim J. Kong W. H. Kim K. S. Kim C. (2019). Multifunctional nanodroplets encapsulating naphthalocyanine and perfluorohexane for bimodal image-guided therapy. Biomacromolecules 20 (10), 3767–3777. 10.1021/acs.biomac.9b00842 31483619

[B8] Choi W. Choi H. Kim J. Kim C. Hahn S. K. (2020) “Surface-crosslinked multi-functional nanodroplets for photoacoustic/ultrasound image-guided high intensity focused ultrasound therapy,” in Photons plus ultrasound: Imaging and sensing 2020 (Bellingham, Washington: International Society for Optics and Photonics), 1124040.

[B9] Cui W. Bei J. Wang S. Zhi G. Zhao Y. Zhou X. (2005). Preparation and evaluation of poly(L-lactide-co-glycolide) (PLGA) microbubbles as a contrast agent for myocardial contrast echocardiography. J. Biomed. Mat. Res. B Appl. Biomater. 73B (1), 171–178. 10.1002/jbm.b.30189 15678494

[B10] Dash T. K. Konkimalla V. B. (2012). Poly-є-caprolactone based formulations for drug delivery and tissue engineering: A review. J. Control. Release 158 (1), 15–33. 10.1016/j.jconrel.2011.09.064 21963774

[B11] Deshpande N. Needles A. Willmann J. K. (2010). Molecular ultrasound imaging: Current status and future directions. Clin. Radiol. 65 (7), 567–581. 10.1016/j.crad.2010.02.013 20541656 PMC3144865

[B12] Díaz-López R. Tsapis N. Fattal E. (2010). Liquid perfluorocarbons as contrast agents for ultrasonography and 19F-mri. Pharm. Res. 27 (1), 1–16. 10.1007/s11095-009-0001-5 19902338

[B13] Duco W. Grosso V. Zaccari D. Soltermann A. T. (2016). Generation of ROS mediated by mechanical waves (ultrasound) and its possible applications. Methods 109, 141–148. 10.1016/j.ymeth.2016.07.015 27542338

[B14] Fang Y. Cheng J. Shen Z. You T. Ding S. Hu J. (2022). Ultrasound‐mediated release of gaseous signaling molecules for biomedical applications. Macromol. Rapid Commun. 43, 2100814. 10.1002/marc.202100814 35032066

[B15] Gorce J.-M. Arditi M. Schneider M. (2000). Influence of bubble size distribution on the echogenicity of ultrasound contrast agents: A study of SonoVue™. Invest. Radiol. 35(11), 661–671. 10.1097/00004424-200011000-00003 11110302

[B16] Hasannia M. Aliabadi A. Abnous K. Taghdisi S. M. Ramezani M. Alibolandi M. (2022). Synthesis of block copolymers used in polymersome fabrication: Application in drug delivery. J. Control. Release 341, 95–117. 10.1016/j.jconrel.2021.11.010 34774891

[B17] Heer H. Kaur V. P. Rathor S. Aamir S. Singh C. (2021). “Microbubbles used for drug delivery system,” in Nanopharmaceutical advanced delivery systems (New Jersey, United States: Wiley), 125–143.

[B18] Houvenagel S. Moine L. Picheth G. Dejean C. Brûlet A. Chennevière A. (2018). Comb-like fluorophilic-lipophilic-hydrophilic polymers for nanocapsules as ultrasound contrast agents. Biomacromolecules 19 (8), 3244–3256. 10.1021/acs.biomac.8b00506 29995383

[B19] Huang H.-Y. Lin C.-A. J. Chang W. H. Yeh C.-K. (2016). Template-based formation of ultrasound microbubble contrast agents. RSC Adv. 6 (73), 69185–69190. 10.1039/C6RA09316G

[B20] Ishihara K. Ueda T. Nakabayashi N. (1990). Preparation of phospholipid polylners and their properties as polymer hydrogel membranes. Polym. J. 22 (5), 355–360. 10.1295/polymj.22.355

[B21] Kee A. L. Y. Teo B. M. (2019). Biomedical applications of acoustically responsive phase shift nanodroplets: Current status and future directions. Ultrason. Sonochem. 56, 37–45. 10.1016/j.ultsonch.2019.03.024 31101274

[B22] Kripfgans O. D. Fabiilli M. L. Carson P. L. Fowlkes J. B. (2004). On the acoustic vaporization of micrometer-sized droplets. J. Acoust. Soc. Am. 116 (1), 272–281. 10.1121/1.1755236 15295987

[B23] Lattin J. R. Belnap D. M. Pitt W. G. (2012). Formation of eLiposomes as a drug delivery vehicle. Colloids Surf. B Biointerfaces 89, 93–100. 10.1016/j.colsurfb.2011.08.030 21962853

[B24] Ma Y. Tang Y. Billingham N. C. Armes S. P. Lewis A. L. Lloyd A. W. (2003). Well-defined biocompatible block copolymers via atom transfer radical polymerization of 2-methacryloyloxyethyl phosphorylcholine in protic media. Macromolecules 36 (10), 3475–3484. 10.1021/ma021762c

[B25] Martinez H. P. Kono Y. Blair S. L. Sandoval S. Wang-Rodriguez J. Mattrey R. F. (2010). Hard shell gas-filled contrast enhancement particles for colour Doppler ultrasound imaging of tumors. MedChemComm 1 (4), 266–270. 10.1039/C0MD00139B 21841967 PMC3155153

[B26] Matyjaszewski K. Dong H. Jakubowski W. Pietrasik J. Kusumo A. (2007). Grafting from surfaces for “everyone”: ARGET ATRP in the presence of air. Langmuir 23 (8), 4528–4531. 10.1021/la063402e 17371060

[B27] McEwan C. Fowley C. Nomikou N. McCaughan B. McHale A. P. Callan J. F. (2014). Polymeric microbubbles as delivery vehicles for sensitizers in sonodynamic therapy. Langmuir 30 (49), 14926–14930. 10.1021/la503929c 25409533

[B28] Mountford P. A. Borden M. A. (2016). On the thermodynamics and kinetics of superheated fluorocarbon phase-change agents. Adv. Colloid Interface Sci. 237, 15–27. 10.1016/j.cis.2016.08.007 27574721

[B29] Mullin L. Gessner R. Kwan J. Kaya M. Borden M. A. Dayton P. A. (2011). Effect of anesthesia carrier gas on *in vivo* circulation times of ultrasound microbubble contrast agents in rats. Contrast Media Mol. Imaging 6 (3), 126–131. 10.1002/cmmi.414 21246710 PMC3341737

[B30] Nyankima A. G. Rojas J. D. Cianciolo R. Johnson K. A. Dayton P. A. (2018). *In vivo* assessment of the potential for renal bio-effects from the vaporization of perfluorocarbon phase-change contrast agents. Ultrasound Med. Biol. 44 (2), 368–376. 10.1016/j.ultrasmedbio.2017.10.016 29254872

[B31] Pang E. H. Chan A. Ho S. G. Harris A. C. (2018). Contrast-enhanced ultrasound of the liver: Optimizing technique and clinical applications. AJR. Am. J. Roentgenol. 210 (2), 320–332. 10.2214/AJR.17.17843 29220210

[B32] Park Y. Luce A. C. Whitaker R. D. Amin B. Cabodi M. Nap R. J. (2012). Tunable diacetylene polymerized shell microbubbles as ultrasound contrast agents. Langmuir 28 (8), 3766–3772. 10.1021/la204510h 22260537 PMC3302155

[B33] Petersen M. A. Hillmyer M. A. Kokkoli E. (2013). Bioresorbable polymersomes for targeted delivery of cisplatin. Bioconjug. Chem. 24 (4), 533–543. 10.1021/bc3003259 23521104

[B34] Qin D. Zhang L. Zhu H. Chen J. Wu D. Bouakaz A. (2021a). A highly efficient one-for-all nanodroplet for ultrasound imaging-guided and cavitation-enhanced photothermal therapy. Int. J. Nanomedicine 16, 3105–3119. 10.2147/IJN.S301734 33967577 PMC8096805

[B35] Qin D. Zhang L. Zhu H. Chen J. Wu D. Bouakaz A. (2021b). A highly efficient one-for-all nanodroplet for ultrasound imaging-guided and cavitation-enhanced photothermal therapy. Int. J. Nanomedicine 16, 3105–3119. 10.2147/IJN.S301734 33967577 PMC8096805

[B36] Quaia E. (2007). Microbubble ultrasound contrast agents: An update. Eur. Radiol. 17 (8), 1995–2008. 10.1007/s00330-007-0623-0 17351779

[B37] Rana D. Matsuura T. (2010). Surface modifications for antifouling membranes. Chem. Rev. 110 (4), 2448–2471. 10.1021/cr800208y 20095575

[B38] Rapoport N. Y. Kennedy A. M. Shea J. E. Scaife C. L. Nam K.-H. (2009). Controlled and targeted tumor chemotherapy by ultrasound-activated nanoemulsions/microbubbles. J. Control. Release 138 (3), 268–276. 10.1016/j.jconrel.2009.05.026 19477208 PMC2746980

[B39] Sanna V. Pintus G. Bandiera P. Anedda R. Punzoni S. Sanna B. (2011). Development of polymeric microbubbles targeted to prostate-specific membrane antigen as prototype of novel ultrasound contrast agents. Mol. Pharm. 8 (3), 748–757. 10.1021/mp100360g 21545176

[B40] Schlenoff J. B. (2014). Zwitteration: Coating surfaces with zwitterionic functionality to reduce nonspecific adsorption. Langmuir 30 (32), 9625–9636. 10.1021/la500057j 24754399 PMC4140545

[B41] Sharma D. Leong K. X. Czarnota G. J. (2022). Application of ultrasound combined with microbubbles for cancer therapy. Int. J. Mol. Sci. 23 (8), 4393. 10.3390/ijms23084393 35457210 PMC9026557

[B42] Sheeran P. S. Luois S. Dayton P. A. Matsunaga T. O. (2011). Formulation and acoustic studies of a new phase-shift agent for diagnostic and therapeutic ultrasound. Langmuir 27 (17), 10412–10420. 10.1021/la2013705 21744860 PMC3164903

[B43] Song S. Guo H. Jiang Z. Jin Y. Zhang Z. Sun K. (2014). Self-assembled Fe3O4/polymer hybrid microbubble with MRI/ultrasound dual-imaging enhancement. Langmuir 30 (35), 10557–10561. 10.1021/la5021115 25136957

[B44] Su C. Ren X. Nie F. Li T. Lv W. Li H. (2021). Current advances in ultrasound-combined nanobubbles for cancer-targeted therapy: A review of the current status and future perspectives. RSC Adv. 11 (21), 12915–12928. 10.1039/D0RA08727K 35423829 PMC8697319

[B45] Sun Y. Zheng Y. Ran H. Zhou Y. Shen H. Chen Y. (2012). Superparamagnetic PLGA-iron oxide microcapsules for dual-modality US/MR imaging and high intensity focused US breast cancer ablation. Biomaterials 33 (24), 5854–5864. 10.1016/j.biomaterials.2012.04.062 22617321

[B46] Tang W. Matyjaszewski K. (2007). Effects of initiator structure on activation rate constants in ATRP. Macromolecules 40 (6), 1858–1863. 10.1021/ma062897b

[B47] Tu S. Chen Y. W. Qiu Y. B. Zhu K. Luo X. L. (2011). Enhancement of cellular uptake and antitumor efficiencies of micelles with phosphorylcholine. Macromol. Biosci. 11 (10), 1416–1425. 10.1002/mabi.201100111 21793214

[B48] Vidallon M. L. P. Giles L. W. Pottage M. J. Butler C. S. G. Crawford S. A. Bishop A. I. (2022). Tracking the heat-triggered phase change of polydopamine-shelled, perfluorocarbon emulsion droplets into microbubbles using neutron scattering. J. Colloid Interface Sci. 607, 836–847. 10.1016/j.jcis.2021.08.162 34536938

[B49] Wang G. Shi Y. Fu Z. Yang W. Huang Q. Zhang Y. (2005). Controlled synthesis of poly (ε-caprolactone)-graft-polystyrene by atom transfer radical polymerization with poly (ε-caprolactone-co-α-bromo-ε-caprolactone) copolymer as macroinitiator. Polymer 46 (23), 10601–10606. 10.1016/j.polymer.2005.06.105

[B50] Wei P. Cornel E. J. Du J. (2021). Ultrasound-responsive polymer-based drug delivery systems. Drug Deliv. Transl. Res. 11 (4), 1323–1339. 10.1007/s13346-021-00963-0 33761101 PMC7989687

[B51] Woodruff M. A. Hutmacher D. W. (2010). The return of a forgotten polymer—polycaprolactone in the 21st century. Prog. Polym. Sci. 35 (10), 1217–1256. 10.1016/j.progpolymsci.2010.04.002

[B52] Xu S. Yang F. Zhou X. Zhuang Y. Liu B. Mu Y. (2015). Uniform PEGylated PLGA microcapsules with embedded Fe3O4 nanoparticles for US/MR dual-modality imaging. ACS Appl. Mat. Interfaces 7 (36), 20460–20468. 10.1021/acsami.5b06594 26327472

[B53] Yang P. Ding J. Guo J. Shi W. Hu J. J. Wang C. (2013). A strategy for fabrication of uniform double-shell hollow microspheres as effective acoustic echo imaging contrast agents through a new polymer-backbone-transition method. J. Mat. Chem. B 1 (4), 544–551. 10.1039/C2TB00059H 32260826

[B54] Yang Y. Achazi K. Jia Y. Wei Q. Haag R. Li J. (2016). Complex assembly of polymer conjugated mesoporous silica nanoparticles for intracellular pH-responsive drug delivery. Langmuir 32 (47), 12453–12460. 10.1021/acs.langmuir.6b01845 27467698

[B55] Yu L. Zhang M. Du F.-S. Li Z.-C. (2018). ROS-responsive poly (ε-caprolactone) with pendent thioether and selenide motifs. Polym. Chem. 9 (27), 3762–3773. 10.1039/c8py00620b

[B56] Zhang C. Wang Z. Wang C. Li X. Liu J. Xu M. (2016). Highly uniform perfluoropropane-loaded cerasomal microbubbles as a novel ultrasound contrast agent. ACS Appl. Mat. Interfaces 8 (24), 15024–15032. 10.1021/acsami.5b03668 26114237

[B57] Zhang P. Cao Y. Chen H. Zhou B. Hu W. Zhang L. (2017). Preparation and evaluation of glycyrrhetinic acid-modified and honokiol-loaded acoustic nanodroplets for targeted tumor imaging and therapy with low-boiling-point phase-change perfluorocarbon. J. Mat. Chem. B 5 (29), 5845–5853. 10.1039/c7tb01215b 32264217

[B58] Zhao L.-Y. Chao X. Yang B.-S. Wang G.-G. Zou J.-Z. Wu F. (2022). Phase-shift perfluoropentane nanoemulsions enhance pulsed high-intensity focused ultrasound ablation in an isolated perfused liver system and their potential value for cancer therapy. J. Ultrasound Med. 41 (1), 107–121. 10.1002/jum.15686 33724514

[B59] Zhou Y. (2015). Application of acoustic droplet vaporization in ultrasound therapy. J. Ther. Ultrasound 3 (1), 20–18. 10.1186/s40349-015-0041-8 26566442 PMC4642755

[B60] Zhu B. Wang L. Huang J. Xiang X. Tang Y. Cheng C. (2019). Ultrasound-triggered perfluorocarbon-derived nanobombs for targeted therapies of rheumatoid arthritis. J. Mat. Chem. B 7, 4581–4591. 10.1039/C9TB00978G

