# Supplementary material for: Phospholipid-mimicking block, graft, and block-graft copolymers for phase-transition microbubbles as ultrasound contrast agents
Source: Front Pharmacol. 2022 Oct 13;13:968835. doi: 10.3389/fphar.2022.968835 (PMC9606805; doi:10.3389/fphar.2022.968835)
Supplement: Supplementary file 1 [file Table1.DOCX]

**Supplementary Material**

**Phospholipid-Mimicking Block, Graft, and Block-Graft Copolymers for** **Phase-Transition Microbubbles** **as Ultrasound Contrast Agents**

Jianbo Huang ^1^, Hong Wang ^1^*, Lei Huang ^2^, Yuqing Zhou ^1^

1. Department of Ultrasound, Laboratory of Ultrasound Medicine, West China Hospital, Sichuan University, Chengdu 610041, China. E-mail: wanghonggjm@163.com;

2. College of Polymer Science and Engineering, State Key Laboratory of Polymer Materials Engineering, Sichuan University, Chengdu 610065, China.

^1^H-NMR data were obtained using a Bruker spectrometer (400 MHz, DRX 300, Germany). The CDCl_3_ and CD_3_OD were used as the solvent and the tetramethyl silane (TMS) was used as the internal standard. The Fourier transform infrared spectroscopy (FTIR) spectra of polymers were acquired on a FTIR spectrometer (Nicolet 560, USA). The gel permeation chromatography (GPC) test was used to determine the molecular weight and distribution of polymers. The THF was used as moving phase and the polystyrene was used as the standard. The flow rate was 0.6 mL/min at 40 °C. The thermal properties of polymers were characterized by differential scanning calorimetry (DSC). The rate was 10 °C/min.

**Differential scanning calorimetry (DSC)**

The thermal properties of the polymer were characterized by DSC. Firstly, dry the polymer sample, then weigh the sample (3 ~ 5 mg) and put it into the aluminum dry pot for sample preparation and put it into the instrument. Take nitrogen as the shielding gas and set the flow rate as 10 ml / min. The temperature was raised from room temperature to 100 °C at a heating rate of 50 °C / min for 3 minutes to eliminate the thermal history of the polymer. Then the temperature was reduced to - 80 °C at the rate of 10 °C / min for 3 min to obtain the crystallization curve of the polymer. Finally, the melting curve of the polymer was obtained by raising the temperature to 100 °C at the rate of 10 °C / min.The crystallinity of the polymer is calculated according to formula:

$$X_{c}=\frac{\Delta H_{f}(T_{m})}{\Delta H_{f}^{0}(T_{m}^{0})}$$

Where $\Delta H_{f}^{0}(T_{m}^{0})$ = 139.3 J/g, which is the melting enthalpy when PCL is fully crystallized, and $\Delta H_{f}(T_{m}^{0})$ is obtained from the melting peak integral of the melting curve, which is the melting enthalpy of the tested polymer.

**Supplementary Table 1. DSC data of polymers**

| **Material** | **T_c_(**^o^C**)** | **T_m_(** ^o^C) | **ΔH_c_(J/g)** | **ΔH_m_(J/g)** | **X_c_(%)** |
| --- | --- | --- | --- | --- | --- |
| PCL_34_ | 32.7 | 54 | 67.4 | 70.3 | 50.47% |
| PCL_40_-*g*-PMPC_5×5_ | 6.6 | 34.3 | 1.9 | 13.7 | 9.83% |
| PCL_34_-*b*-(PBrCL_5_-*g*-PMPC_5×5_) | 20.2 | 46.9 | 5.1 | 16.7 | 11.99% |
| PCL_43_-b-PMPC_25_ | 22.1 | 52.2 | 7.8 | 26 | 18.66% |

**Supplementary Table 2. Characterization of PCL and PCL-PMPC copolymer**

| **Polymers** | **M_n_^a^** | **M_n_^b^** | **M_w_/M_n_^b^** | **Theoretical content (mol%)^b^** | | | **Elemental analysis(mol%)^c^** | | |
| --- | --- | --- | --- | --- | --- | --- | --- | --- | --- |
|  |  |  |  | **C** | **O** | **P** | **C** | **O** | **P** |
| PCL_34_ | 4060 | 6100 | 1.15 | - | - | - | - | - | - |
| PCL_34_-b-PBCL_5_ | 5030 | 11200 | 1.35 | - | - | - | - | - | - |
| PCL_43_-Br | 5090 | 13800 | 1.44 | - | - | - | - | - | - |
| PCL_40_-*g*-PMPC_5×5_ | 12540 | - | - | 66.88 | 29.87 | 3.25 | 68.46 | 29.43 | 2.11 |
| PCL_34_-*b*-(PBrCL_5_-*g*-PMPC_5×5_) | 12410 | - | - | 66.80 | 29.92 | 3.28 | 67.47 | 30.19 | 2.34 |
| PCL_43_-b-PMPC_25_ | 12470 | - | - | 67.13 | 29.72 | 3.15 | 70.19 | 27.74 | 2.07 |

^a^ Calculated by ^1^H-NMR peak area ratio; ^b^ Calculated by GPC; ^c^ Calculated from Energy Dispersive Spectrometer; “-” means the values were not measured.


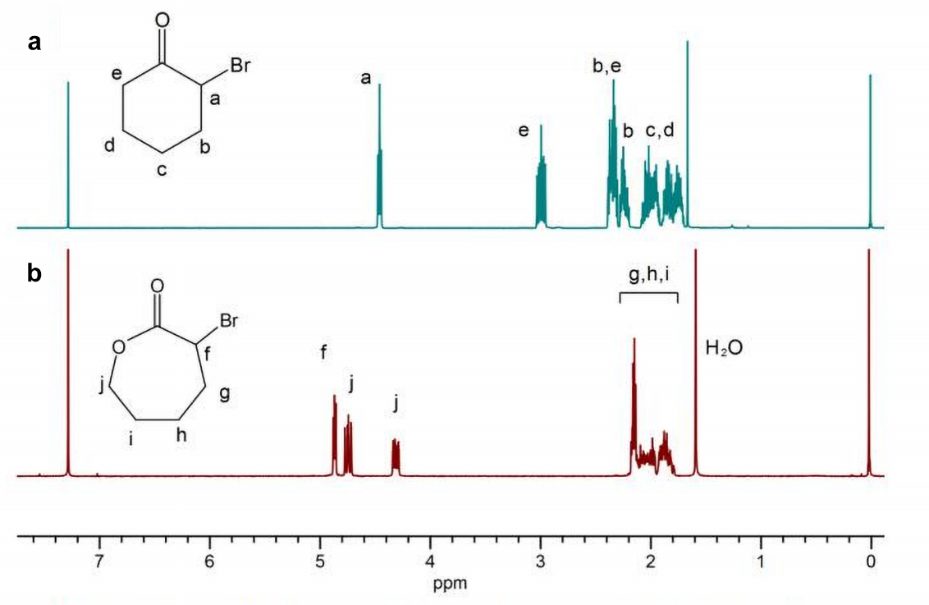


Supplementary Figure 1. 400 M ^1^H NMR spectra of BCHO (a) and αBrεCL (b) in CDCl_3_

Stannous octanoate was an efficient ring opening polymerization catalyst, which was widely used in ring opening polymerization of εCL. In addition, the structural similarity ofαBrεCL and εCL indicated that they may have the same polymerization mechanism. Therefore, we used stannous octanoate as catalyst and lauryl alcohol as initiator, inducing αBrεCL and εCL ring opening polymerization to synthesize P (BCL-co-CL) random copolymer. The 400 M ^1^H NMR spectrum of P (BCL-co-CL) was shown in Figure S1.


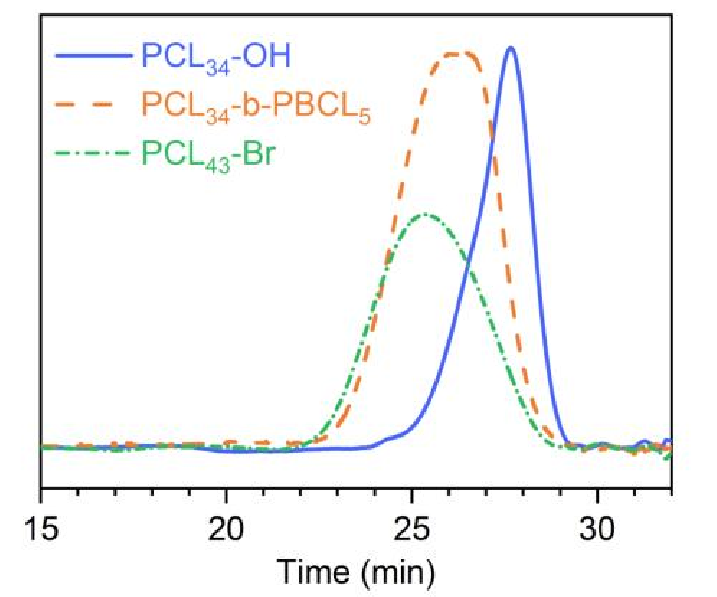


Supplementary Figure 2. GPC curves of PCL_34_, PCL_34_-b-PBCL_5_ and PCL_43_-Br.

The molecular weight and polydispersity of PCL and PCL-b-PBCL were measured by GPC, and the elemental composition of C, O and P in the copolymer of PCL-g-PMPC was measured by EDS. The results were shown in Figure S2 and Table S2. It could be seen from Figure S2 that the GPC curves of PCL_34_, PCL_34_-B-PBCL_5_ and PCL_43_-Br were single-peak distribution, and the flow time of PCL_34_-b-PBCL_5_ was smaller than that of PCL_34_, indicating that the ring-opening polymerization of αBrεCL of PCL_34_ was successful. At the same time, the outflow time of PCL_43_-Br was similar to that of PCL_34_-OH, which indicates that the relative molecular weight of both was approximate. The element composition measured by EDS was consistent with that calculated by NMR, which indicated that the synthesis of PCL-PMPC was successful.


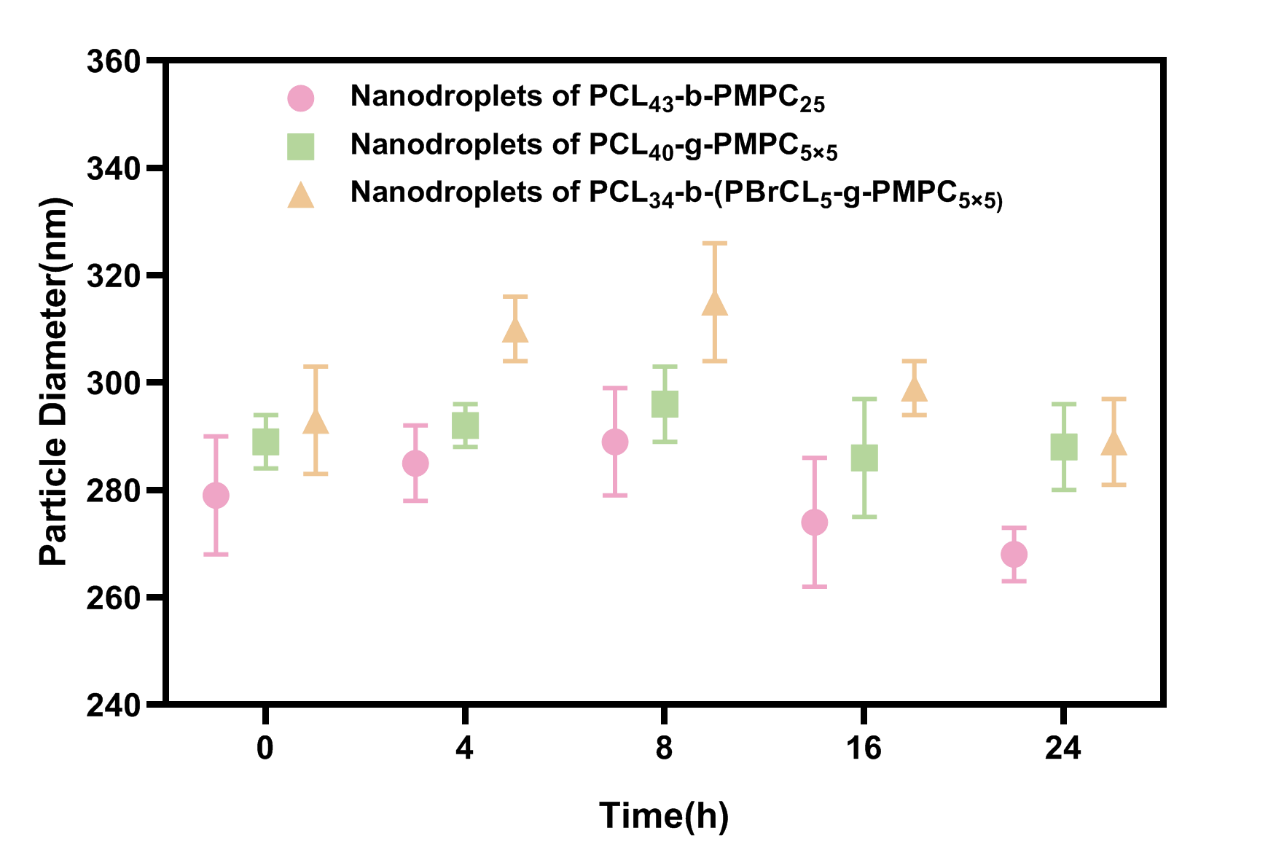


Supplementary Figure 3. Particle Diameter of these three nanodroplets during 24 hours at 37 °C.


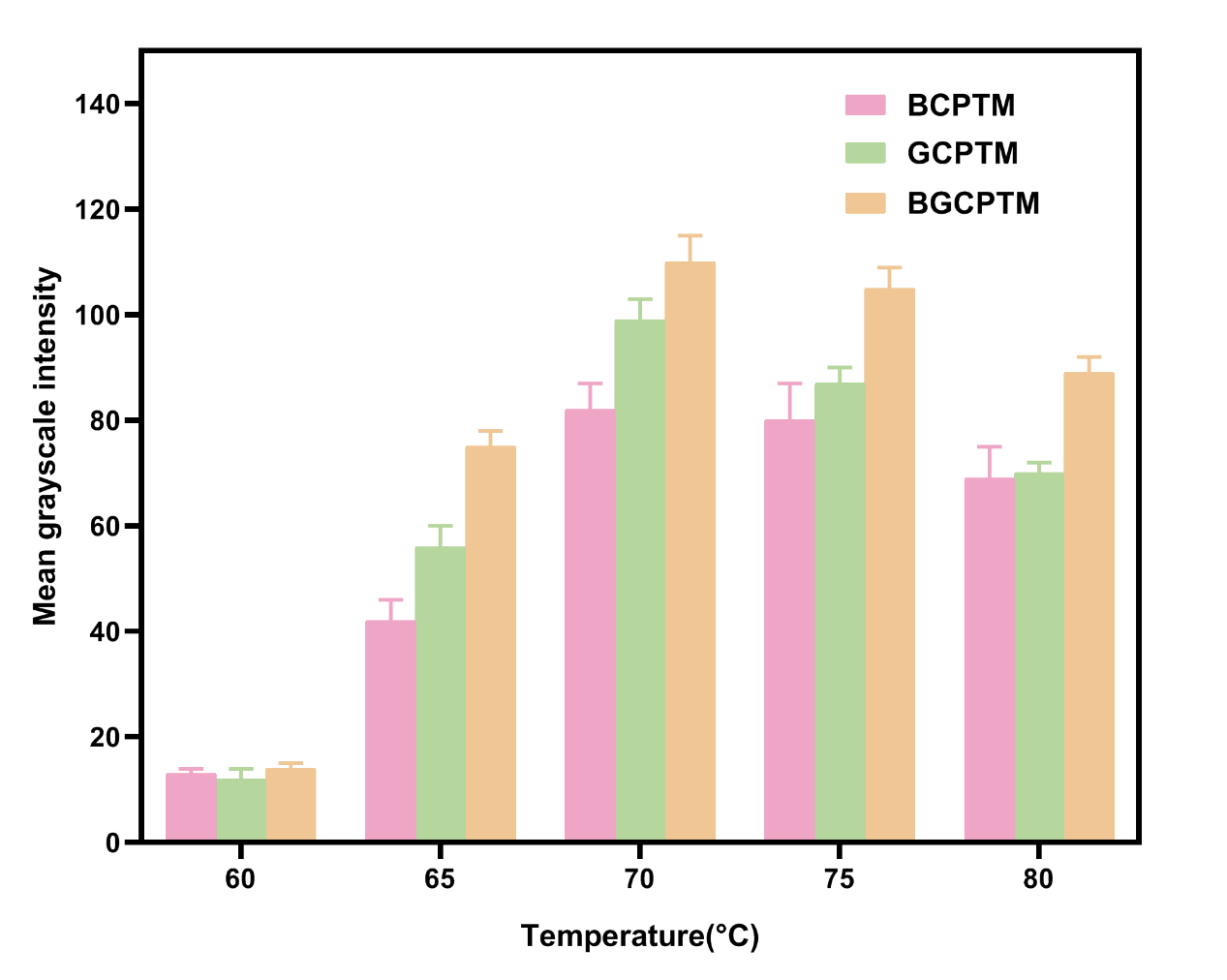


Supplementary Figure 4. Mean grayscale intensity of the obtained CEUS images under different BCPTM, GCPTM and BGCPTM under different temperature of water bath 10 mins .
